# Predictive value of infiltrating tumor border configuration of rectal cancer on MRI

**DOI:** 10.1186/s12880-023-01118-y

**Published:** 2023-10-12

**Authors:** Baohua Lv, Leilei Yuan, Jizheng Li, Xue Kong, Yanling Cheng, Kai Shang, Erhu Jin

**Affiliations:** 1 Department of Radiology, Taian City Central Hospital, Qingdao University, Tai’an, 271099 China; 2 Department of Radiology, Beijing Friendship Hospital, Capital Medical University, No. 95, Yong-an Road, Beijing, 100050 China; 3 Respiratory department of Shandong Second Rehabilitation Hospital, Tai’an, 271000 China; 4 Department of Orthopedic, Taian City Central Hospital, Qingdao University, Tai’an, 271099 China

**Keywords:** Magnetic resonance imaging, Extramural vascular invasion, Rectal cancer, Tumor border configuration

## Abstract

**Background:**

Infiltrating tumor border configuration (iTBC) is assessed by postoperative pathological examination, thus, is not helpful for preoperative treatment strategies. The study aimed to detect iTBC by magnetic resonance imaging (MRI) and evaluate its predictive value.

**Materials and methods:**

A total of 153 patients with rectal cancer were retrospectively analyzed. Clinicopathological and MRI data mainly including tumor border configuration (TBC) on MRI, MRI-detected extramural vascular invasion (MEMVI), tumor length, tumor growth pattern, maximal extramural depth, pathology-proven lymph node metastasis (PLN) and pathology-proven extramural vascular invasion (PEMVI) were analyzed. The correlation of MRI factors with PEMVI and PLN was analyzed by univariate and multivariate logistic regression analyses. The nomograms were established based on multivariate logistic regression analysis and were confirmed by Bootstrap self-sampling. The receiver operating characteristic (ROC) curve analysis and area under the curve (AUC) were used to evaluate the diagnostic efficiency.

**Results:**

Fifty cases of PEMVI and 48 cases of PLN were found. Forty cases of PEMVI and 34 cases of PLN in 62 cases of iTBC were also found. iTBC, MEMVI and maximal extramural depth were significantly associated with PEMVI and PLN (*P* < 0.05). iTBC (odds ratio = 3.84 and 3.02) and MEMVI (odds ratio = 7.27 and 3.22) were independent risk factors for PEMVI and PLN. The C-indices of the two nomograms for predicting PEMVI and PLN were 0.863 and 0.752, respectively. The calibration curves and ROC curves of the two nomograms showed that the correlation between the predicted and the actual incidence of PEMVI and PLN was good. The AUCs of iTBC for predicting PEMVI and PLN were 0.793 (95% CI: 0.714–0.872) and 0.721 (95% CI: 0.632–0.810), respectively. The DeLong test showed that the predictive efficiency of the nomogram in predicting PEMVI was better than that of iTBC (*P* = 0.0009) and MEMVI (*P* = 0.0095).

**Conclusion:**

iTBC and MEMVI are risk factors for PEMVI and pelvic lymph node metastasis. The nomograms based on iTBC show a good performance in predicting PEMVI and pelvic lymph node metastasis, possessing a certain clinical reference value.

**Trial registration:**

This study was approved by the Ethics Committee of Beijing Friendship Hospital, and individual consent was waived for this retrospective analysis.

## Background

Colorectal cancer is one of the four deadliest cancers in the world, with the incidence ranking third and mortality ranking second. In 2020, more than 1.9 million patients were newly diagnosed with colorectal cancer worldwide, and 940,000 people died of rectal cancer [1]. Colorectal cancer incidence is on the rise in developing countries, and the number of new colorectal cancer cases is expected to increase to 2.5 million globally by 2035 [2, 3]. Tumor stage, grade of tumor differentiation, circumferential margin status, lymph node metastasis, venous infiltration and perineural infiltration are all important factors affecting the prognosis of patients [4–8]. Recently, a number of pathologically related studies found that TBC had an impact on the prognosis of rectal cancer patients, particularly in patients with iTBC, which had poor overall survival and disease-free survival and was significantly associated with several pathological parameters of poor prognosis [9, 10]. The concept of TBC in histopathology was first proposed by Jass et al. in 1987, and Jass divided the mass margin morphology into iTBC and pushing TBC (pTBC) [11, 12]. However, these are obtained after surgery, which is not helpful for the formulation of the preoperative treatment plan. In our daily work, we found that some of the marginal morphology of primary rectal cancer tumors observed on MRI may correspond to the histopathologically clustered or fascicular neoplasia of iTBC, and these morphology on MRI were often associated with extramural vascular invasion (EMVI) [13], lymph node metastasis and distant metastasis. Moreover, the study of MRI about TBC has not been reported, so our study aimed to detect TBC by MRI and evaluate its predictive value.

## Materials and methods

### Patients

**Fig. 1:**
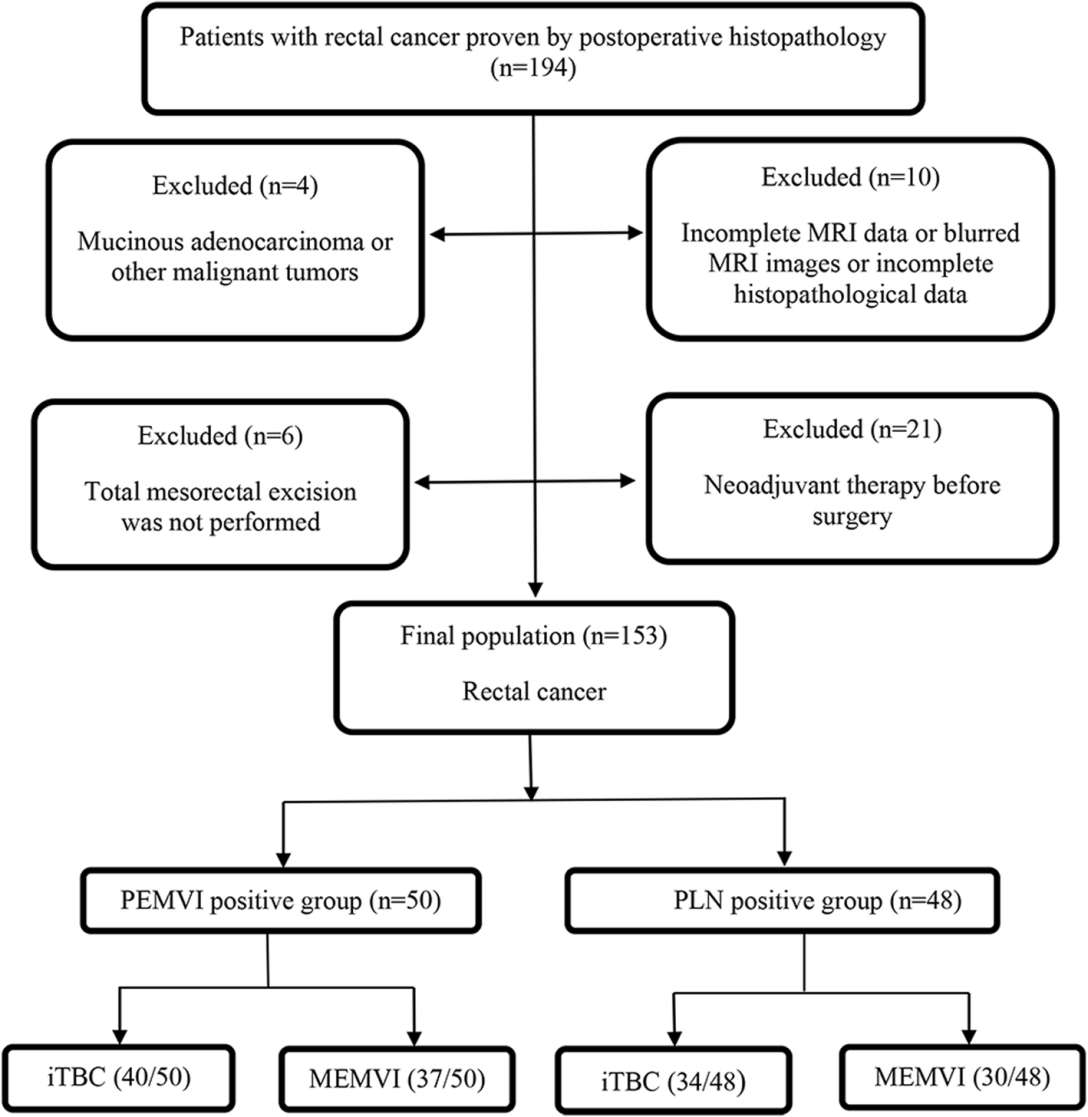
Study flow diagram. PEMVI: pathology-proven extramural vascular invasion; PLN: pathology-proven lymph node involvement; iTBC: infiltrating tumor border configuration; MEMVI: MRI-detected extramural vascular invasion

The medical records and MRI data of 194 patients in our hospital from 2014 to 2019 were retrospectively analyzed. The rectal cancer of all patients was confirmed by postoperative histopathology. All MRI scans of rectal cancer patients were done before neoadjuvant therapy or surgery. The exclusion criteria included: (i) MRI sequence was incomplete or image quality was blurry, which affected the assessment of MRI signs in this study; (ii) patient without total mesorectal excision; (iii) patient who had preoperative neoadjuvant therapy; (iv) patient with incomplete histopathological data; (v) patient with mucinous adenocarcinoma for their specific biological behavior or with other malignant diseases. Finally, 153 cases were enrolled (Fig. 1).

### MRI technique

MRI was performed using a 3.0 T system (Signa Excite HD 3.0 T, GE Healthcare, Milwaukee, WI, USA) equipped with an 8-channel body surface coil. Patients were advised to eat light food 24 h before the scan according to the daily volume and defecate in time. Before the MR scan, the rectum was cleaned with an enema, and no antispasmodic drugs were administered during the examination. Pulse sequences were observed by fast spin-echo sagittal high-resolution T2-weighted imaging (HRT2WI) with a thickness of 3 mm, an intersection gap of 0.5 mm, and a TR/TE of 4,000 ms/102 ms, in the absence of fat saturation. The matrix size was 384 × 360. The echo train length (ETL) and number of excitations (NEX) was 16 and 4, respectively. The oblique axial HRT2WI was perpendicular to the rectal wall and covered the entire tumor in the absence of fat saturation with a thickness of 3 mm, an intersection gap of 0.5 mm, a TR/TE of 4,900 ms/96 ms, a matrix size 320X256, field of view (FOV) 20-22 cm and resolution ratio 0.87mmX0.78 mm. When oblique coronal HRT2WI was performed, the scanning plane was parallel to the intestinal wall of the lesion area and covers the whole tumor. A LAVA/ LAVA-XV sequence was performed for dynamic contrast-enhanced MRI in the presence of fat saturation, a thickness of 3 mm, FOV of 36 × 36 cm, matrix size of 256 × 192, flip angle of 15°, and 40 consecutive phases. Gadolinium-diethylenetriaminepentaacetic acid (Gd-DTPA) (0.1 mmol/kg, Magnevist, Bayer Schering, Germany) was intravenously injected at a rate of 2 ml/s, followed by a saline flush before enhanced sequencing.

### Imaging interpretation

The MRI images of all patients were retrospectively analyzed on oblique axial, sagittal, oblique coronal HRT2WI images and gadolinium-enhanced T1WI by two radiologists with 10 and 15 years of experience in abdominal MRI and familiar with the diagnostic criteria. In case of disagreement, a third experienced gastrointestinal radiologist was involved and consensus was reached by discussion. All radiologists were blind to the pathological results of EMVI and lymph node.

#### Evaluation of multiple MRI signs

**Fig. 2:**
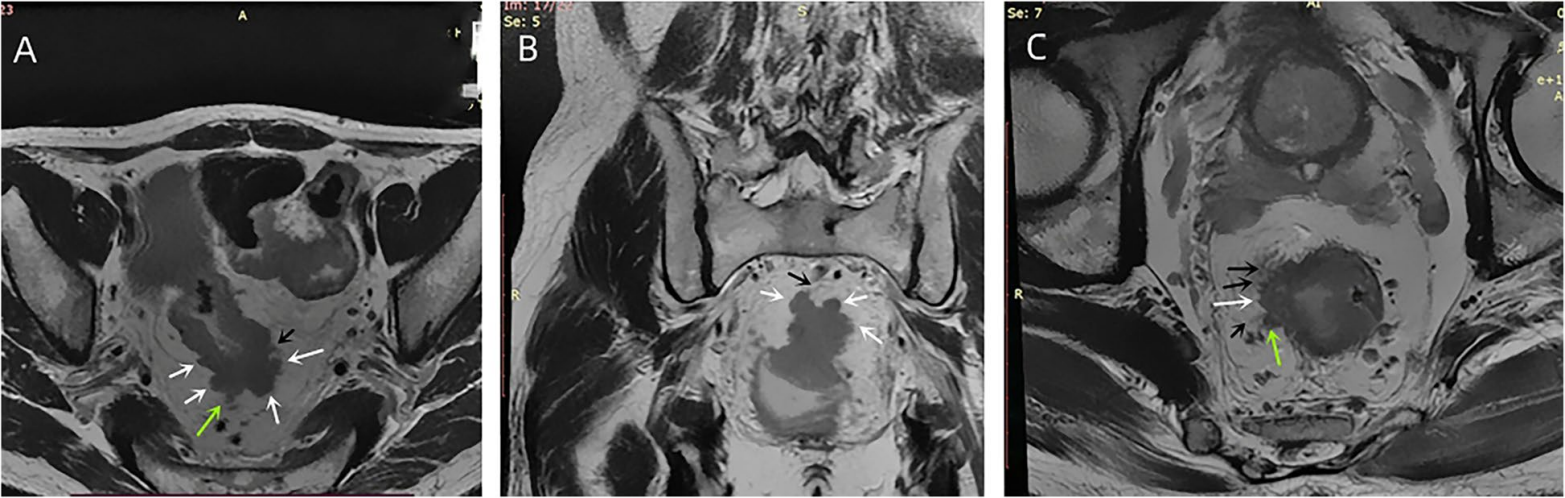
Nodular protrusions of iTBC. Oblique axial (**A**) and oblique coronal (**B**) HRT2WI images of a patient with rectal cancer (PT3N1) showed multiple irregular nodular protrusions (white arrows) appeared at the margin of the primary tumor. Some nodules had lobulated edges (green arrows), and some nodules had irregular cords (black arrows). Oblique axial HRT2WI (C) image of another patient with rectal cancer (PT3N1) showed the primary rectal tumor had a large nodule protruding (white arrow) at the right margin, and this nodule protrusion had small nodules (green arrow) and multiple irregular cords (multiple black arrows) at its edge

**Fig. 3:**
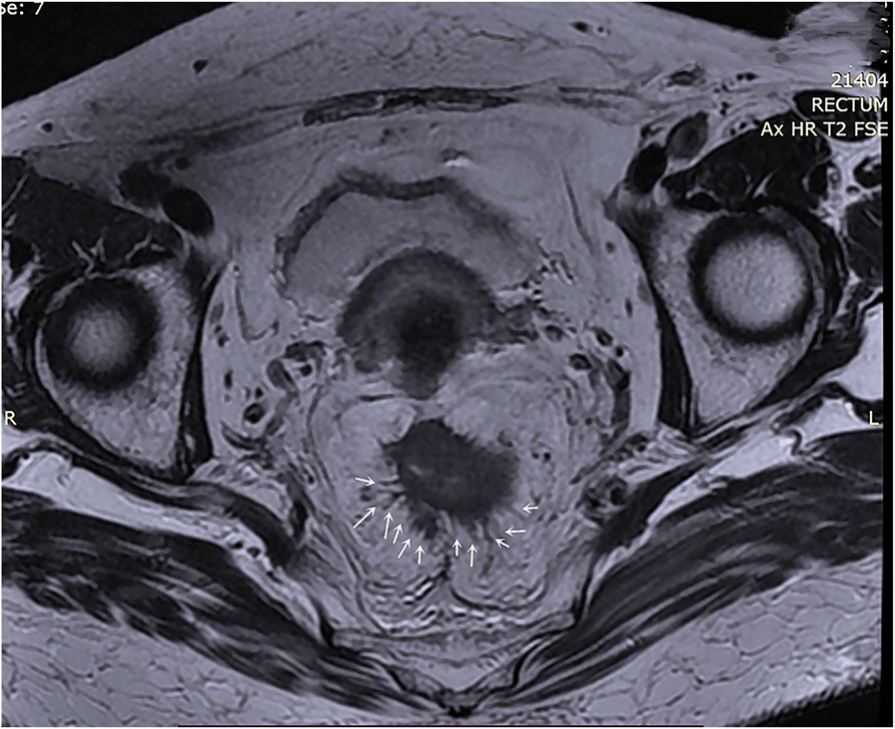
Multiple cords of iTBC. Oblique axial HRT2WI image of a patient with rectal cancer (PT3N1) showed there were multiple cords (multiple white arrows) of uneven thickness at the margin of the primary rectal tumor

Referring to the histopathological TBC, we also divided the MRI tumor border morphology into iTBC and pTBC. iTBC was mainly characterized by the following aspects: (1) the presence of multiple nodular protrusions of different sizes at the margin of the primary tumor (Fig. 2A and B); (2) if the tumor had a single nodular protrusion at the margin, the nodule had an irregular morphology, which also had a hairy and lobulated margin or multiple cords of uneven thickness at its margin (Fig. 2C); (3) presence of multiple cords rough and not smooth of uneven thickness on the edge of the primary tumor (Fig. 3). pTBC was mainly characterized by the following aspects: the margins of the primary tumor or the single nodular protrusions at the tumor margin were clear and smooth, or the cords at the tumor margin were uniformly thick and well-defined.

MEMVI was evaluated according to Smith's five-point rating system [14]. (1) score 0: the tumor did not penetrate the muscularis propria of the rectal wall. (2) score 1: the tumor invaded the muscularis propria of the rectal wall, but no blood vessels were found around the tumor. (3) score 2: the tumor invaded the muscularis propria of the rectal wall, but the diameter and signal of the peritumoral blood vessels was normal, and no abnormal blood vessels were observed in gadolinium-enhanced T1WI. (4) score 3: the tumor invaded the muscularis propria of the rectal wall and intestinal wall, and the peritumoral vascular diameter was slightly irregularly dilated, or the tumor tissue with moderate intensity signal was observed in the lumen by gadolinium-enhanced T1WI; (5) score 4: the tumor penetrated the muscularis propria of the rectum wall, and the lumen of one or more blood vessels around the tumor were irregularly expanded. Tumor tissue was present in the lumen, and the signal intensity was moderate. A significant irregular dilation of the lumen of one or more vessels in the tumor was observed, with tumor-like enhancement of the abnormal signals in their lumens was found by gadolinium-enhanced T1WI. The MEMVI negative had a score of 0 to 2, while the MEMVI positive had a score of 3 to 4.

#### Evaluation of other MRI parameters

**Fig. 4:**
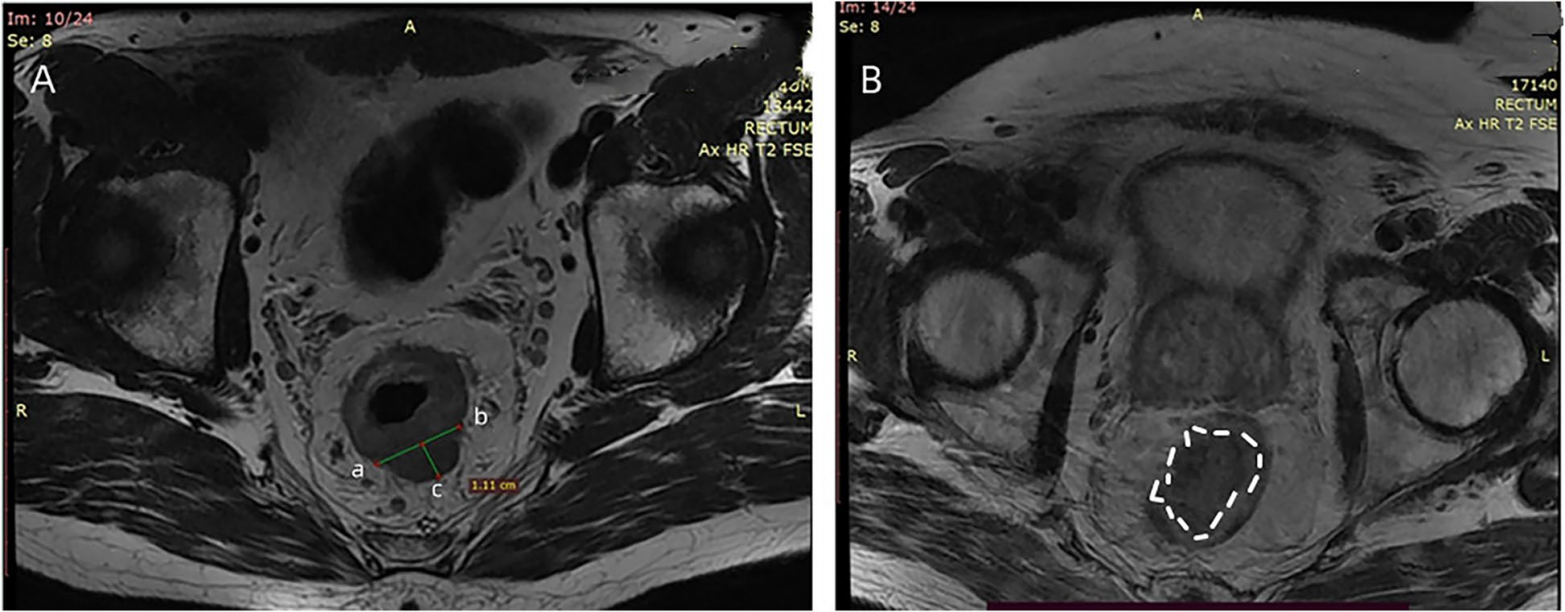
Growth pattern of tumor and the measurement of maximal extramural depth. **A**: on oblique axial HRT2WI, the rectal tumor invaded the entire rectal wall, showing a complete annular shape. The maximal extramural depth was the distance between the attachment in the two muscularis propria breakthroughs and the outermost boundary of the tumor (1.11 cm). **B**: a local mass appeared in the right wall of the rectum (white dotted circle), with less than one-half of the invaded rectal wall

The length from the most marginal tumor to the rectal muscularis propria on the oblique axial HRT2WI was defined as maximal extramural depth and was divided into two groups: < 5 mm and ≥ 5 mm. When the recal muscularis propria was not identified on the oblique axial HRT2WI, the vertical distance from the most distal part of the tumor to the line between the residual muscularis propria on both tumorous sides was measured (Fig. 4A) [15]. Tumor length was the length measured at multiple points along the sagittal axis of the diseased bowel between the upper and lower tumorous borders and was divided into two groups: < 5 cm and ≥ 5 cm. The tumor growth pattern was mainly circular infiltration and local mass. Circular infiltration was defined as annular growth of rectal tumor along the rectal wall on oblique axial HRT2WI, with more than half of the rectal wall invaded, and the primary rectal tumor was annular or semi-annular (Fig. 4A). Local mass was defined as a rounded or oval mass with less than half of the rectal wall affected on oblique axial HRT2WI (Fig. 4B). The distance between the lower margin of rectal tumor and the anal margin was more than 5 cm, which was classified as middle and upper rectal cancer, and less than 5 cm was classified as lower rectal cancer. The range of the rectal wall involved in the tumor was estimated on oblique axial HRT2WI and was divided into three groups: ≤ 1/3, 1/3–2/3 and ≥ 2/3.

### Clinicopathological assessment

Postoperatively, specimens were soaked in 10% formalin for more than 48 h and sectioned laterally perpendicular to the long axis of the rectum with a thickness of 3 µm. Tumor differentiation, involved lymph nodes and EMVI were collected and analyzed by histopathological examination. EMVI was confirmed by histopathology when the tumor tissue existed in the extramural space of the wall or in tubular structures arranged by endothelial cells, smooth muscle or elastic fibers [16]. Histopathology was performed by a pathologist with extensive experience in colorectal pathology.

### Statistical analysis

Statistical analysis was performed using BM SPSS 26.0, MedCalc and R (R 4.2.1) software. The correlation between clinicopathological and MRI factors with PEMVI and PLN weas analyzed by Chi-square test or ANOVA. The statistically significant variables from univariate analysis were assessed by multivariate logistic regression analysis to identify independent predictors of PEMVI and PLN. The nomograms for predicting PEMVI and PLN were constructed on basis of multivariate logistic regression analysis. The predictive diagnostic performance of iTBC and MEMVI for PEMVI and PLN was evaluated by plotting the ROC curve. Comparisons of ROC curves between MRI indicators and nomograms were performed by the DeLong test. Agreements between pathological findings and imaging assessment results were performed using the Kappa test (Kappa > 0.75 indicates good consistency, 0.40 > Kappa ≤ 0.75 indicates moderate consistency, and Kappa ≤ 0.40 indicates poor consistency). A value of *P* < 0.05 was considered statistically significant.

## Results

### Patient characteristics

A total of 153 rectal cancer patients (100 males and 53 females) were enrolled in this study. The mean age of all patients was 63.7 ± 10.7 yrs. The mean age of male patients was 64.4 ± 10.6 yrs and the mean age of female patients was 62.5 ± 10.9 yrs. Demographic and MRI data are listed in Table 1.

**Table 1:** Characteristics of MRI and clinicopathological data

Variables	*n* = 153	PEMVI			PLN		
		Negative (*n* = 103) n (%)	Positive (*n* = 50) n (%)	*P* value	Negative (*n* = 105) n (%)	Positive (*n* = 48) n (%)	*P* value
Age (year)	63.69 ± 10.68	64.19 ± 11.12	62.84 ± 9.90	0.466	64.77 ± 10.61	61.52 ± 10.74	0.082
Gender				0.908			0.615
Male	100 (65.4)	67 (65.0)	33 (66.0)		70(66.7)	30(62.5)	
Female	53 (34.6)	36 (35.0)	17 (34.0)		35(33.3)	18(37.5)	
Range of rectal wall invasion				0.505			0.04
≤ 1/3	11 (7.1)	9 (8.7)	2 (4.0)		10(9.5)	1(2.1)	
1/3–2/3	55 (35.3)	38 (36.9)	17(34.0)		42(40.0)	13(27.1)	
≥ 2/3	87 (56.9)	56 (54.4)	31 (62.0)		53(50.5)	34(70.8)	
TBC				<0.001			<0.001
iTBC	62(40.5)	22(21.4)	40(80.0)		28(26.7)	34(70.8)	
pTBC	91(59.5)	81(78.6)	10(20.0)		77(73.3)	14(29.2)	
MEMVI				<0.001			<0.001
Positive	50 (32.7)	13 (12.6)	37 (74.0)		20(19.0)	30(62.5)	\
Negative	103(67.3)	90(87.4)	13(36.0)		85(81.0)	18(37.5)	
Growth pattern				0.382			0.021
Local mass	72 (47.1)	51 (49.5)	21 (42.0)		56(53.3)	16(33.3)	
Circular infiltration	81 (52.9)	52 (50.5)	29 (58.0)		49(46.7)	32(66.7)	
Tumor location				0.624			0.06
Upper-middle	108 (70.6)	74 (71.8)	34 (68.0)		69(65.7)	39(81.3)	
Lower	45 (29.4)	29 (28.2)	16 (32.0)		36(34.3)	9(18.7)	
Tumor length				0.578			0.43
≥ 5 cm	63(41.2)	44(42.7)	19(38.0)		41(39.0)	22(45.8)	
< 5 cm	90(78.8)	59(57.3)	31(62.0)		64(61.0)	26(54.2)	
PLN				<0.001			
Positive	48 (31.4)	18 (17.5)	30 (60.0)				
Negative	105(68.6)	85(82.5)	20(40.0)				
Differentiation				0.191			0.046
Well	8(5.2)	7(6.7)	1(2.1)		7(6.7)	1(2.1)	
Moderately	129(84.3)	88(85.4)	41(82.0)		91(86.7)	38(79.2)	
Poorly	16(10.5)	8(7.8)	8(16.0)		7(6.7)	9(18.8)	
Maximal extramural depth				<0.001			<0.001
≥ 5 mm	54(35.3)	20(19.4)	34(68.0)		23(21.9)	31(64.6)	
< 5 mm	99(64.7)	83(80.6)	16(32.0)		82(78.1)	17(35.4)	

*PEMVI* Pathology-proven extramural vascular invasion, *MEMVI* MRI-detected extramural vascular invasion, *TBC* Tumor border configuration, *iTBC* Infiltrating tumor border configuration, *pTBC* Pushing tumor border configuration, *PLN* Pathology-proven local node involvement

### Pathological results

Among the 153 patients, 50 (50/153, 32.7%) were positive for EMVI according to pathological results, and the other 103 cases were PEMVI negative. 30 (30/48, 62.5%) of 48 PLN were from PEMVI positive group and 18 (18/48, 37.5%) of which were from PEMVI negative group. PLN was associated with poor tumor differentiation. Other pathological results are listed in Table 1.

### Results of univariate analysis

iTBC, MEMVI and maximal extramural depth were significantly correlated with PEMVI and PLN (*P* < 0.001). Rectal cancer patients with circular infiltration and large rectal wall involvement were more likely to develop pelvic lymph node metastasis. The maximal extramural depth of PEMVI-positive and PLN-positive groups were greater than that in the correspondent negative groups (*P* < 0.001). There were no significant differences in age, sex, tumor location and tumor length between the positive and negative groups of PEMVI and PLN. Sixty-two (62/153, 40.5%) cases of iTBC were found, among them 40 (40/62, 64.5%) developed PEMVI and 34 (34/62, 54.8%) developed PLN. Fifty (50/153, 32.7%) cases of MEMVI were found, among them 37 (37/50, 74.0%) developed PEMVI and 30 (30/50, 60.0%) developed PLN. MEMVI and PEMVI had moderate agreement (Kappa = 0.614).

### Multivariate logistic regression analysis and nomograms for PEMVI and PLN

**Fig. 5:**
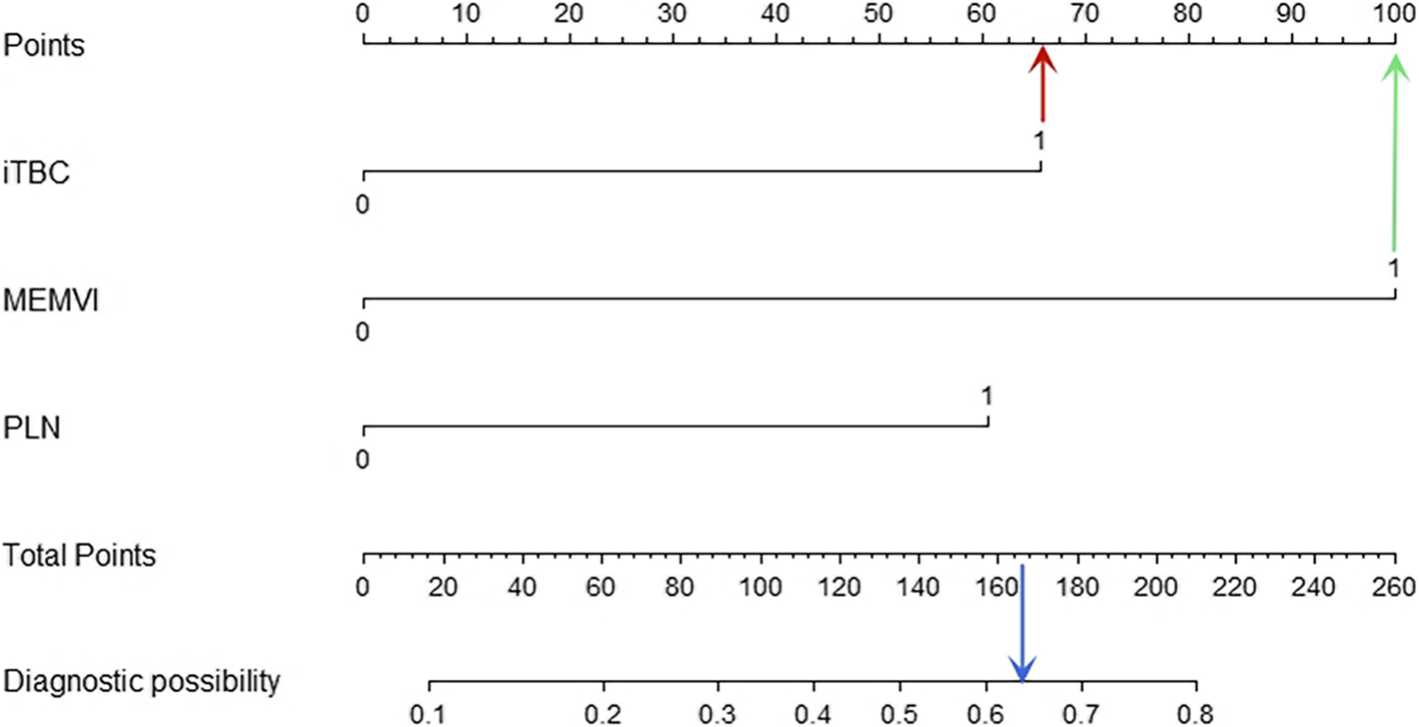
Nomogram for predicting PEMVI. A total score of 166 (66 + 100) for a patient with rectal cancer subjected to infiltrative tumor border configuration (iTBC) (red arrow) and MRI-detected extramural vascular invasion (MEMVI) (green arrow) would place the patient at approximately 64% risk of developing EMVI (blue arrow)

**Fig. 6:**
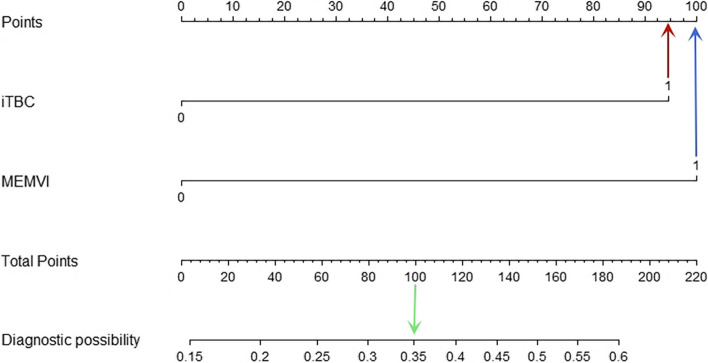
Nomogram for predicting PLN. The risk of developing PLN of a patient with rectal cancer subjected only to MRI-detected extramural vascular invasion (MEMVI) (blue arrow) and total points of 100 was approximately 35% (green arrow)

**Fig. 7:**
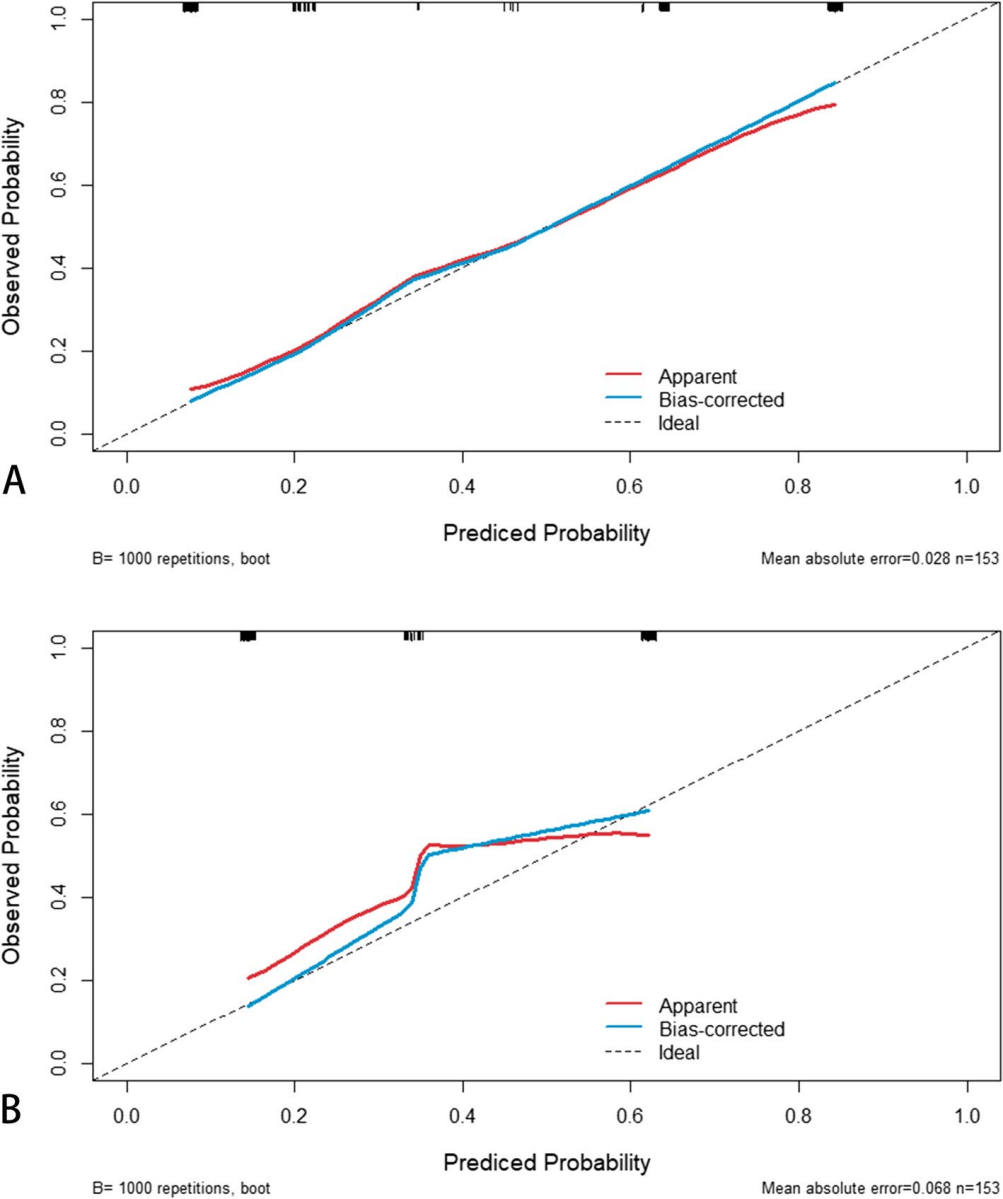
Calibration curve of nomogram for predicting PEMVI (**A**) and PLN (**B**). The x-axis represents the nomogram-predicted probability and the y-axis represents the actual probability of PEMVI (**A**) and PLN (**B**). The perfect prediction corresponds to the 45° dotted line. The red solid line represents the entire cohort (*n* = 153), and the blue solid line is bias-corrected by bootstrapping (B = 1000 repetitons), indicating the observed nomogram performance. The calibration curve showed an evident relationship between the actual tag and the predicted tag

**Fig. 8:**
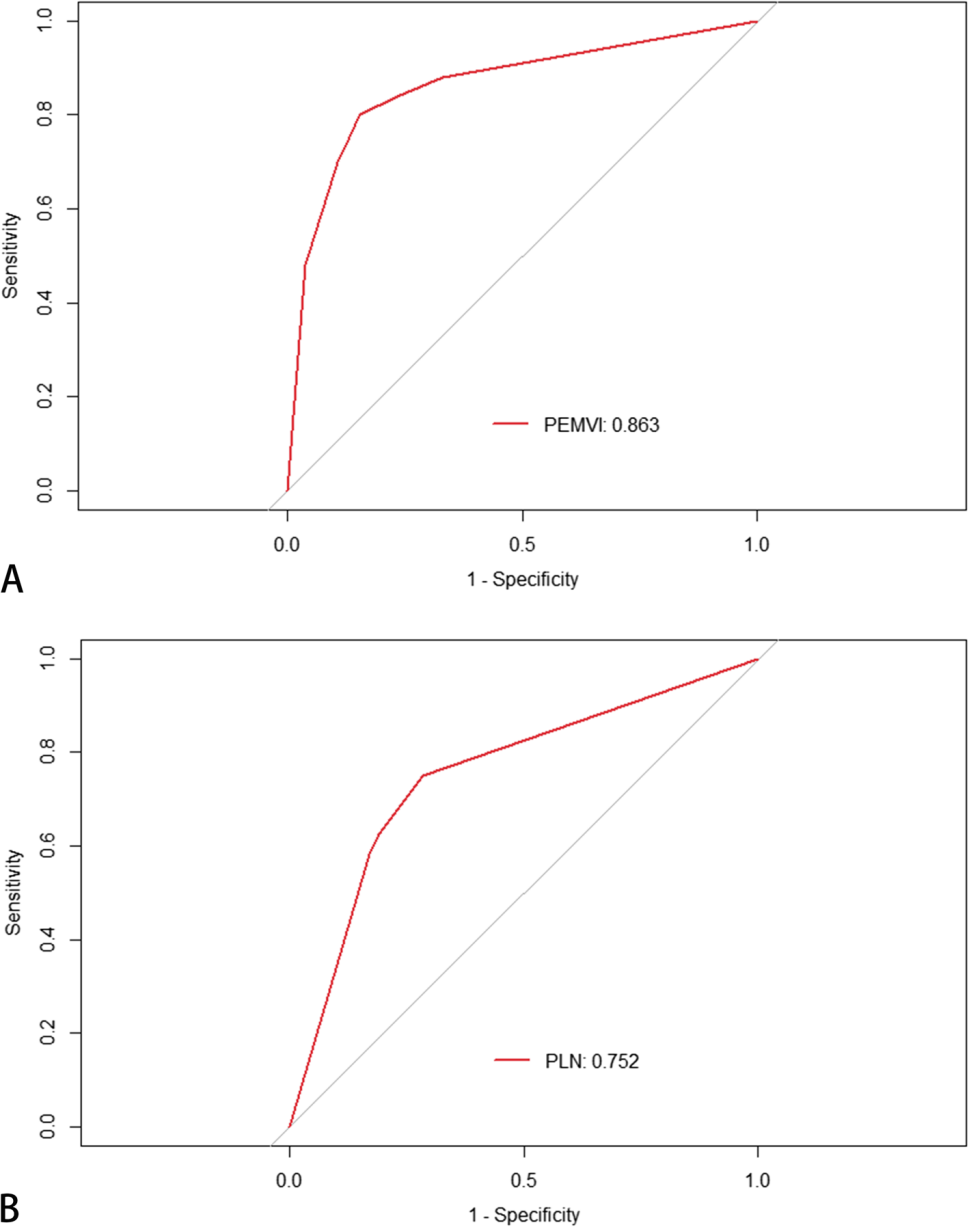
ROC curves of nomograms for predicting PEMVI (**A**) and PLN (**B**). The areas under ROC curves of nomograms for predicting PEMVI (**A**) and PLN (**B**) were 0.863 and 0.752, respectively. This suggested that the confidence level of the probability of EMVI and pelvic lymph node metastasis by the two nomograms was 86.3% and 75.2%, respectively

Multivariate logistic regression analysis showed that iTBC, MEMVI and PLN were independent predictors for PEMVI, with odds ratios of 3.84, 7.27 and 3.67, respectively. MEMVI and iTBC were independent predictors for PLN with odds ratios of 3.22 and 3.02, respectively (Table 2). Based on the results of multivariate logistic regression analysis, two nomograms were constructed to predict PEMVI and PLN (Figs. 5 and 6). After internal verification by bootstrap self-sampling, the C-indices of two nomograms were 0.863 and 0.752, respectively. The calibration curves (Fig. 7) and ROC curves (Fig. 8) of the two nomograms showed that the predicted probability was well correlated with their actual incidence.

**Table 2:** Results of multivariate logistic regression analysis

Independent predictor	PEMVI			PLN		
	Odds ratio	95% CI	*P* value	Odds ratio	95% CI	*P* value
iTBC	3.84	1.17–12.59	0.026	3.02	1.06–8.57	0.038
MEMVI	7.27	2.25–23.48	<0.001	3.22	1.13–9.12	0.028
PLN	3.67	1.37–9.93	0.010			

*PEMVI* Pathology-proven extramural vascular invasion, *PLN* Pathology-proven lymph node involvement, *iTBC* infiltrating tumor border configuration, *MEMVI* MRI-detected extramural vascular invasion

### Predictive performance of MRI indicators and nomograms for PEMVI and PLN

**Fig. 9:**
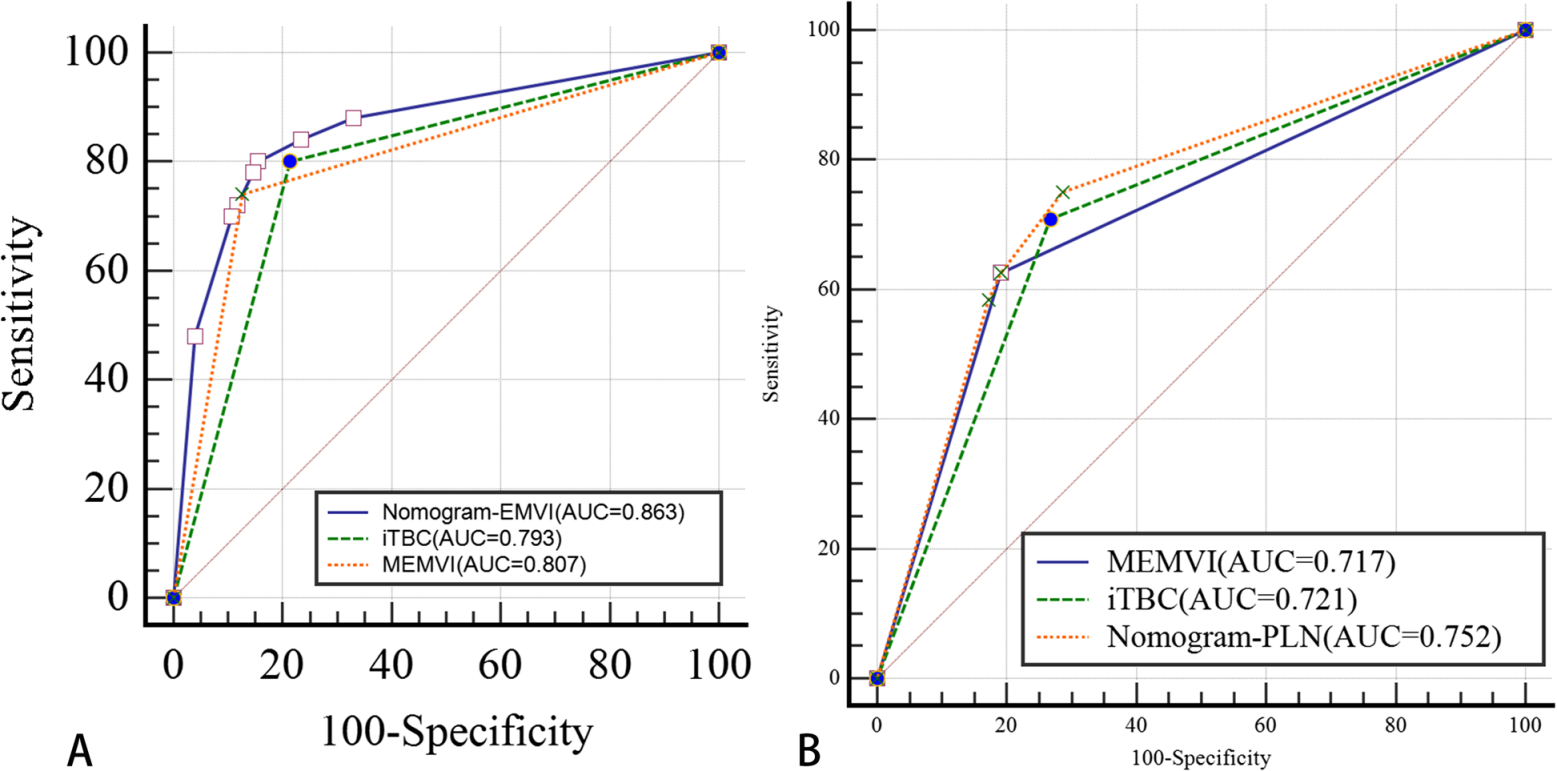
Comparison of ROC curves for predicting PEMVI (**A**) and PLN (**B**). iTBC: infiltrating tumor border configuration; MEMVI: MRI-detected extramural vascular invasion

The AUC of iTBC for predicting PEMVI was 0.793 (95% CI: 0.714–0.872), with a sensitivity of 80.0%, a specificity of 78.6%, a positive predictive value (PPV) of 64.5%, a negative predictive value (NPV) of 89.0%, and an accuracy of 79.1% (Table 3). The AUC of iTBC for predicting PLN was 0.721 (95% CI: 0.632-0.810),  with a sensitivity of 70.8%, a specificity of 73.3%, a PPV of 54.9%, an NPV of 84.6%, and an accuracy of 72.5% (Table 3). The DeLong test showed no statistically significant difference between the ROC curves of iTBC and MEMVI in predicting PEMVI (*P* = 0.663). The AUC of the nomogram for predicting PEMVI was 0.863 (95% CI: 0.798–0.928) (Fig. 8), which was statistically different from iTBC (AUC = 0.793, *P* = 0.0009) and MEMVI (AUC = 0.807, *P* = 0.0095) (Fig. 9), with a sensitivity of 80.0%, a specificity of 84.5%, a PPV of 71.4%, a NPV of 89.7%, and an accuracy of 83.0%, respectively (Table 3). The AUC of the nomogram for predicting PLN was not statistically different from iTBC (*P* = 0.0873) and MEMVI (*P* = 0.0745) (Fig. 9).

**Table 3:** Diagnostic predictive values in predicting PEMVI and PLN

Model	Predictive factor	Sensitivity (%)	Specificity (%)	PPV (%)	NPV (%)	Accuracy (%)	AUC (95% CI)
PEMVI	MEMVI	74.0	87.4	74.0	87.4	69.9	0.807(0.726–0.888)
	Maximal extramural depth (≥ 5 mm)	68.0	80.6	63.0	83.8	76.5	0.743(0.655–0.831)
	iTBC	80.0	78.6	64.5	89.0	79.1	0.793(0.714–0.872)
	Nomogram (EMVI)	80.0	84.5	71.4	89.7	83.0	0.863(0.798–0.928)
PLN	MEMVI	62.5	81.0	60.0	82.5	76.5	0.717(0.625–0.810)
	Maximal extramural depth (≥ 5 mm)	64.6	78.1	57.4	82.8	73.9	0.713(0.622–0.805)
	iTBC	70.8	73.3	54.9	84.6	72.5	0.721(0.632–0.810)
	Nomogram (PLN)	75.0	71.4	54.5	86.2	72.5	0.752(0.673–0.831)

*PPV* Positive predictive value, *NPV* Negative predictive value, *AUC* Area under the curve, *PEMVI* Pathology-proven extramural vascular invasion, *MEMVI* MRI-detected extramural vascular invasion, *iTBC* Infiltrating tumor border configuration, *PLN* Pathology-proven lymph node metastasis

## Discussion

According to Jass's research, iTBC was defined as malignant tumor penetrated the intestinal wall in irregular clusters or cords and had a diminished inflammatory response around the tumor [11, 12]. In our daily work, we found that the presence of some tumorous edge forms (e.g. marginal nodal protrusions and cords) with unclear boundary were often accompanied by EMVI, distant metastasis and pelvic lymph node metastasis. Therefore, we speculated that these morphologies of tumor margin might be consistent with histopathological iTBC of JASS. Therefore, we defined iTBC as multiple irregular nodular protrusions and irregular cords at the edge of the tumor observed on MRI, and the boundaries between these nodules or cords and the surrounding adipose tissue were blurred. Clusters and bundles of the iTBC in the study of JASS might be consistent with the nodular protrusions and cords of the iTBC on MRI in our study.

Both Qwaider and Aboelnasr found that iTBC was significantly associated with T stage, lymph node metastasis, and EMVI. They also discovered that iTBC had a negative impact on overall survival and disease-free survival, regardless of other clinical, pathological, and molecular factors [9, 10]. Our study's findings align with theirs, demonstrating the significant association between iTBC and PEMVI and PLN. This suggests that the presence of iTBC observed on MRI corresponds to histopathological findings. Logistic regression analysis further revealed that iTBC independently predicted PEMVI and lymph node metastasis. Patients with iTBC in rectal cancer exhibited a 3.84-fold increased risk of EMVI and a 3.02-fold increased risk of lymph node metastasis compared to those with pTBC. In another study by Halvorsen, patients with iTBC did not exhibit evident peritumoral inflammation [17]. Conversely, pTBC showed a dense inflammatory infiltrate in the peritumoral region. The density of the inflammatory response surrounding the tumor reflects the effectiveness of the anti-tumor host response, which may contribute to why TBC affects patient prognosis [18].

The expression of VEGF and β-catenin in tumor cells at the tumor edge was higher compared to other parts of the tumor [19]. This promotes active tumor cell proliferation at the tumor margin, leading to abundant new blood vessels and rapid growth. Consequently, marginal nodules and cable strips form, increasing tumor volume and facilitating its invasion of extramural vessels. Moreover, we had demonstrated in a recent study that nodular protrusion at the edge of the tumor was significantly associated with EMVI [13]. Rectal cancer can cause desmoplastic reaction, which is the formation of ribbon-like fibrous tissue around the tumor [20]. In particular, cancer-associated fibroblasts formed during desmoplastic reaction were related to tumor dedifferentiation, which can induce tumor budding, promote the transformation of tumor epithelial cells, accelerate tumor invasion, and further enhance the invasiveness of tumor itself. Therefore, some studies had reported that the value of desmoplastic reaction classification as a prognostic parameter may exceed that of tumor tissue differentiation, vascular invasion and tumor stage [21, 22]. To sum up, the presence of nodular protrusion and cords at the tumor margins may suggest poor prognosis in patients with this type of rectal cancer.

Vascular invasion was considered to be the first  step of  hematogenous metastasis. Many studies had shown that PEMVI and MEMVI were closely associated with adverse prognostic events such as local recurrence, distant metastasis, and tumor-related death of rectal cancer [6, 7, 14, 23–25]. Our study also confirmed that MEMVI was a risk factor for pelvic lymph node metastasis, and patients with rectal cancer who developed MEMVI had a 3.22-fold increased risk of pelvic lymph node metastasis, compared with its negative counterpart. At present, high-resolution MRI based on five-level scoring system was the main method for preoperative EMVI detection, but the detectable rate of many studies about MEMVI varied greatly, ranging from 30%-60%, and the sensitivity ranged from 28.2%-62.0% [14, 24, 26, 27]. The reasons for the wide variation in these results may be related to the different pathological histological methods, equipment and MRI parameters, as well as the different levels of awareness of EMVI among pathologists and radiologists, and MRI for pipe diameter less than 3 mm of the small blood vessels invaded to distinguish ability is very limited. Moreover, when blood vessels invaded structure is completely destroyed, can resulting in a false negative of PEMVI. In this study, the positivity rate of MEMVI was only 32.7%, which is relatively low. Apart from the aforementioned factors, this could also be attributed to the lack of utilizing DWI in combination for identifying EMVI. Due to the limitations in slice thickness and resolution, DWI may face difficulties in identifying small vascular lesions. However, DWI has a higher reference value for suspicious intraluminal lesions observed on HRT2WI. The combined application of DWI, HRT2WI, and CET1WI can enhance the detection rate and diagnostic accuracy of EMVI. Specifically, DWI plays a critical role in identifying EMVI after neoadjuvant therapy [28]. In addition to the observation of the vessel itself, other indirect MRI signs should also be analyzed in depth. Our study found that iTBC was significantly correlated with PEMVI. The AUC of iTBC and MEMVI was very close to each other in the predicting PEMVI, and DeLong test showed that there was no statistical difference between them (0.793 VS 0.807, *P* = 0.663), and the sensitivity of iTBC was higher than MEMVI (80.0% vs 74.0%). Compared with MEMVI, the nomogram constructed on the basis of iTBC also showed better predictive performance for the diagnostic accuracy of PEMVI, and helped to screen high-risk patients with EMVI.

At present, a reliable imaging technique for predicting preoperative pelvic lymph node metastasis has not been developed to guide the treatment of patients [29]. The most commonly used criterion for predicting node status is still nodule size, but its performance is not satisfactory [29, 30].  In addition, some researchers have attempted to predict node status by evaluating functional magnetic spectra ranging from diffusion-weighted imaging to dynamic enhancement imaging. However, due to the small size of some of the positive lymph nodes and the low spatial resolution of the images, these studies did not achieve better results [31, 32]. In this study, we constructed nomogram based on iTBC and MEMVI for predicting pelvic lymph node metastasis, which also showed good predictive performance. Therefore, iTBC may be an important MRI sign of EMVI and pelvic lymph node metastasis, which is a useful complement to improve their detection rate. In conclusion, even in the era of molecular pathology and molecular imaging, special attention to tumor morphology is still very important, and combining different tumor morphological parameters and tumor environment can help to obtain more information about tumor behavior and prognosis [33]. Therefore, for rectal cancer patients with iTBC, radiologists should pay special attention to the presence of EMVI and pelvic lymph node metastasis. By utilizing HRT2WI, dynamic contrast-enhanced scanning in combination with DWI, a thorough evaluation of suspicious lesions can be performed to reduce the chance of misdiagnosis. Clinicians should categorize these patients as high-risk individuals for EMVI and pelvic lymph node metastasis. If EMVI or lymph node metastasis is detected, intensified treatment strategies and personalized approaches such as neoadjuvant therapy and postoperative radiation should be adopted. Additionally, it is recommended to shorten the follow-up period, closely monitor changes in the patient's condition, and utilize this information to promptly adjust treatment plans and evaluate therapeutic efficacy. By implementing these measures, rectal cancer patients with such characteristics may potentially benefit more.

Our study also found that maximal extramural depth was statistically significantly correlated with PEMVI and PLN. Because the deeper the tumor infiltration, the more aggressive the tumor is, the larger the tumor volume is, the more the tumor contacts the surrounding vessels and lymph nodes, the higher the chance of vascular and lymph node involvement, and the higher the incidence of hematogenous and lymphogenous metastases [34], In addition, when the tumor is large, it often invades the surrounding organ tissues and rectal mesenteric fascia, which makes it difficult to completely remove the rectal tumor. Our study also found that large range of rectal wall invaded and circular infiltration were prone to regional lymph node metastasis, which may be similar to maximal extramural depth and correlated with large tumor size.

Indeed, this work has also some limitations. Firstly, because of a retrospective study, iTBC was detected by MRI, which was not detected by histopathology in our hospital, and we were unable to accurately compare MRI with histopathology. Secondly, A small part of MEMVI was unequivocal on MRI, but its histopathology showed negative, which may be because the vascular structure was completely destroyed, leading to the failure of histopathologists to identify the vascular structure, resulting in false negative. Thirdly, T stage and the status of the circumferential resection margin were not included in our study for analysis. Lastly, the absence of utilizing DWI in conjunction for identifying EMVI may have contributed to a certain extent in reducing the positivity rate and accuracy of MEMVI.

In conclusion, iTBC on MRI may be used as an independent predictor for PEMVI and PLN. The nomogram based on iTBC has a good predictive value for PEMVI and PLN. These findings facilitate the preoperative selection of patients with high risk of EMVI and pelvic lymph node metastasis based on MRI. For these patients, appropriate treatment strategy and follow-up examination could improve the prognosis as personalized treatment.

## Data Availability

The datasets used and/or analysed during the current study are available from the corresponding author on reasonable request.

## Abbreviations

MRI: Magnetic resonance imaging
EMVI: Extramural vascular invasion
PEMVI: Pathology-proven extramural vascular invasion
MEMVI: MRI-detected extramural vascular invasion
TBC: Tumor border configuration
iTBC: Infiltrating tumor border configuration
pTBC: Pushing tumor border configuration
PLN: Pathology-proven lymph node metastasis
ROC: Receiver operating characteristic
AUC: Area under the curve
HRT2WI: High resolution T2-weighted imaging
ETL: Echo train length
NEX: Number of excitations
FOV: Field of view
Gd-DTPA: Gadolinium-diethylenetriamine pentaacetic acid
PPV: Positive predictive value
NPV: Negative predictive value
